# Nanomaterials for Cardiac Myocyte Tissue Engineering

**DOI:** 10.3390/nano6070133

**Published:** 2016-07-19

**Authors:** Rodolfo Amezcua, Ajay Shirolkar, Carolyn Fraze, David A. Stout

**Affiliations:** 1 Department of Mechanical and Aerospace Engineering, California State University, Long Beach, Long Beach, CA 90840, USA; rodolfo.amez@gmail.com (R.A.); shirolkar.ajay234@gmail.com (A.S.); 2 Deparment of Mechanical Engineering, Brigham Young University-Idaho, Rexburg, ID 83460, USA; fraze.carolyn@gmail.com; 3 Department of Biomedical Engineering, California State University, Long Beach, Long Beach, CA 90840, USA; 4 International Research Center for Translational Orthopaedics, Soochow University, Suzhou 215006, China

**Keywords:** tissue engineering, nanomaterials, cardiac infarction, scaffold, patch, injectable

## Abstract

Since their synthesizing introduction to the research community, nanomaterials have infiltrated almost every corner of science and engineering. Over the last decade, one such field has begun to look at using nanomaterials for beneficial applications in tissue engineering, specifically, cardiac tissue engineering. During a myocardial infarction, part of the cardiac muscle, or myocardium, is deprived of blood. Therefore, the lack of oxygen destroys cardiomyocytes, leaving dead tissue and possibly resulting in the development of arrhythmia, ventricular remodeling, and eventual heart failure. Scarred cardiac muscle results in heart failure for millions of heart attack survivors worldwide. Modern cardiac tissue engineering research has developed nanomaterial applications to combat heart failure, preserve normal heart tissue, and grow healthy myocardium around the infarcted area. This review will discuss the recent progress of nanomaterials for cardiovascular tissue engineering applications through three main nanomaterial approaches: scaffold designs, patches, and injectable materials.

## 1. Introduction

Cardiovascular disease (CVD) is a major epidemic in America and throughout the world. It is the cause of over 800,000 annual deaths in America and 17 million worldwide [1]. Often times, CVD is the main cause of myocardial infarction, the death of cardiomyocytes (CMs) due to a restriction of blood supply to the surrounding myocardium [2]. These CMs, or specialized muscle cells, are necessary in efficiently pumping blood from the heart to the body by contraction-relaxation cycles [3]. As a result of cardiomyocyte apoptosis, myofibroblasts and macrophages migrate towards this area for cardiac repair. These cells then create scar tissue that reduces the contractile ability of the heart, ultimately leading to heart failure [4].

Eventually, when patients arrive at end-stage heart failure, the only effective treatment is heart transplantation or a durable left ventricular assist device (LVAD); however, due to the scarce supply of donor hearts, few patients are fortunate enough to receive transplantation treatment. Furthermore, LVADs, although having drastically improved over the last decade, are associated with bleeding and thrombosis risk and do not address right-sided heart failure [5].

To address this problem, current research has been directed towards cell-based therapies [6] and tissue engineering [7]. The former attempts to use the capability of embryonic stem cells to differentiate into cardiomyocytes, thereby treating the disease. For a review of cell-based therapies, the following articles are recommended [8,9,10]. Tissue engineering is an interdisciplinary field that aims to create biomimetic materials to generate scaffolds usually seeded with cells to either produce or repair functional organs. Cardiac myocyte tissue engineering specifically seeks to create contractile heart muscle tissue for cardiac repair. Early attempts have demonstrated success [11,12]; however, it has become apparent that exploiting the superior material characteristics of nanotechnology has shown the most promising results.

**Figure 1:**
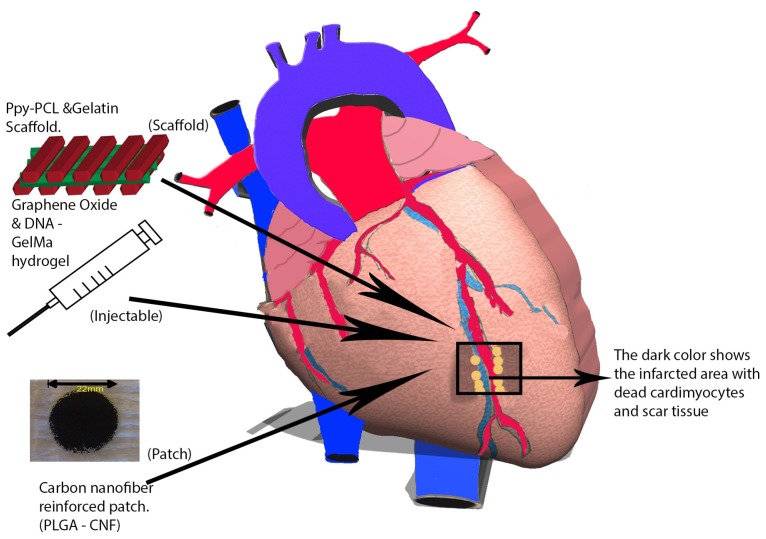
During a myocardial infarction, blood flow is deprived to the myocardium, which, in turn, injures the tissues downstream of the obstruction. As a result, cardiomyocytes are deprived of oxygen and eventually perish. Next, myofibroblasts migrate towards this infarct area and ultimately create scar tissue that reduces the heart’s contractile ability and weakens it. The weakened area is unable to withstand the pressure and volume load on the heart, thereby causing left ventricular dilation. Over time, this scar tissue causes ongoing remodeling, and the heart becomes more spherical, thus impairing systolic and diastolic function. To combat the progress of cardiovascular disease (CVD) and ventricular remodeling, modern research has deployed the use of nanomaterials through the use of scaffolds, patches, and injectable materials for regenerating the heart tissue.

Nanomaterials are commonly made with metals, ceramics, polymers, organic materials, or composites; they consist of nanoparticles, nanoclusters, nanocrystals, nanotubes, or nanofibers [13]. In addition, a variety of techniques are available for their fabrication such as electrospinning, phase separation, self-assembly processes, or thin film deposition [14]. Because these materials are fabricated at the nanoscale, their surface-area-to-volume ratio and surface roughness significantly increases, leading to enhanced mechanical, electrical, optical, catalytic, and magnetic properties [15]. This review will discuss the recent progress of nanomaterials for cardiac myocyte tissue engineering applications through the three commonly used approaches: scaffolds, patches, and injectable materials (Figure 1).

## 2. Scaffolds

The natural extracellular matrix (ECM) is an exceedingly complex material that hosts both structural and regulatory proteins to form a fibrous matrix, and plays an extraordinary role in providing cells with both mechanical and biochemical cues for them to carry out their regular functions [16]. Instead of recreating the ECM, researchers are attempting to fabricate biomimetic scaffolds that provide seeded structural support and guide tissue regeneration. There is also an increasing popularity in using electrically conductive materials such as particular synthetic materials, carbon, or gold to assist CMs in achieving adequate contraction-relaxation cycles. The ideal scaffold is a 3D structure that allows for appropriate mass transfer, is preferably biodegradable at a predetermined rate, offers the necessary surface chemistry for cell adhesion, proliferation, and differentiation, has mechanical/electrical properties that mimic the native tissue, and can be manipulated into various shapes or sizes [17]. Various homogeneous and composite materials have been used to create these scaffolds, many of which incorporate nanoscale features and nanomaterials to enhance their material properties.

### 2.1. Synthetic Materials

**Figure 2:**
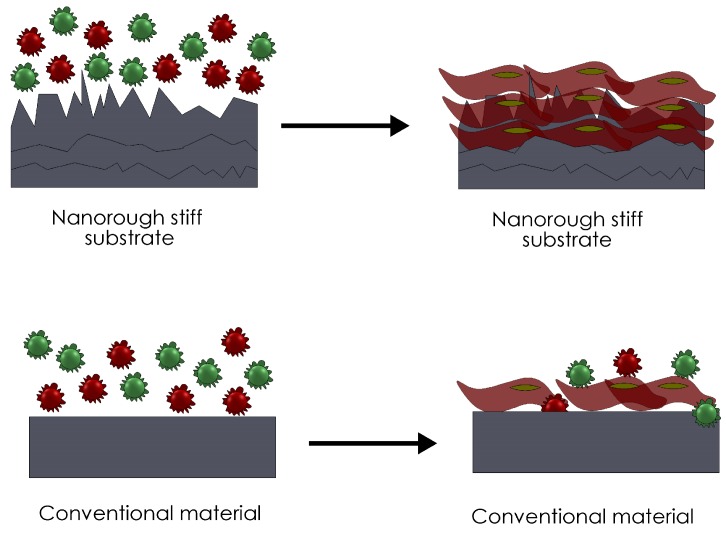
Researchers have exploited nanomaterial characteristics for biomaterial applications by increasing its surface-area ratio. This augmentation in surface-area ratio can enhance protein absorption, thus increasing the possibility of cell recruitment and attachment.

Synthetic materials are commonly used for scaffolding due to their facile fabrication and widely documented mechanical properties. Developing scaffolds with adequate elastic modulus is vital for cardiac tissue engineering because the environment must promote CM contraction. However, the drawback of using synthetic materials, particularly polyester-based thermoplastic polymers is their plastic deformation and failure under long-term cyclic strain [18]. Moreover, due to their low hydrophilicity, synthetic polymers lack surface cell recognition sites, thus cell affinity towards these materials decreases [19]. To work around this problem, research suggests that by simply creating nano-scale topography on the material, cell viability drastically increases (Figure 2).

Poly(ethylene glycol) (PEG) is approved by the Food and Drug Administration (FDA), non-immunogenic material regularly used for scaffolds [20,21]. Kim et al. [22] used PEG to create a substrate with nanopillar topography and found an increase in CM adhesion as compared to conventional PEG substrates. Globular morphology of the CMs was also observed only on the nanostructured substrate, suggesting preferential cell-cell binding resulting from the low surface stiffness of the nanopillars. After investigating the myocardial tissue structure ex vivo and seeing how cell orientation correlates to the ECM fiber alignment, another study used a PEG hydrogel to determine if CMs could be guided by the nanotopography embedded on the substrate [23]. It resulted that the PEG substrate with patterned nanotopography allowed for uniform CM orientation as compared to random orientation on unpatterned nanotopography substrates. Equally important, the study observed larger cell dimensions on the patterned substrate, thus suggesting that nanotopographic patterns influence cell size.

Polyglycolic acid (PGA) is another material approved for biomedical applications and offers desirable degradation rates all while being nontoxic, biocompatible, and hydrophilic [24]. Polycaprolactone (PCL) is a promising candidate for scaffolding because it is FDA approved, is low-cost, but also has soft and flexible characteristics [25,26]. For these reasons, Aghdam et al. [27] used PCL and PGA to construct a synthetic nanofibrous scaffold to culture cardiac progenitor cells. They found cell viability was highest after six days of culture on the synthetic scaffold with 65:35 PCL:PGA weight ratio, and attribute this result to increased hydrophilic properties.

Poly(lactic-co-glycolic) acid (PLGA) is a copolymer of poly-lactic acid (PLA) and PGA. By combining these two materials, PLGA has advantages such as adjustable mechanical properties by controlling its polymer molecular weight, favorable degradation rate, and can be manipulated into any shape and size [28]. For these reasons, PLGA has been extensively used for biological applications. Simon-Yarza et al. used PLGA to create a nanofibrous scaffold to encapsulate recombinant human neuregulin-1 beta isoform (Nrg), a cardiovascular growth factor [29]. This growth factor is known to promote cell recruitment, vasculogensis, and CM replication [30]. Their scaffold was implanted into a myocardial infarcted rat model and successfully integrated into the cardiac tissue. Simon-Yarza et al. [29] also report an increase in M2:M1 macrophage ratio as evidence of constructive remodeling of the infarct zone due to their PLGA scaffold.

Synthetic polymers can also be categorized by their conductivity; Polypyrrole (PPy) is one organic polymer of this class. PPy has been widely investigated and employed for induced neuronal differential of PC-12 cells [31], substrates enhancing endothelial cell proliferation [32], and has been shown to be non-cytotoxic [33]. However, due to its brittle nature and difficulty of mechanical manipulation, it is commonly enhanced with an additive to make it more suitable for cardiac tissue engineering [34]. Kai et al. used PPy in conjunction with PCL and gelatin—a naturally derived material discussed in a later section—to create a mechanically and electrically robust scaffold [35]. PCL was used as an additive because of its ductile and elastic properties. By using a nanofibrous scaffold made from these materials, Kai et al. suggest that the favorable cell proliferation and adhesion on this scaffold could be explained by the formation of electrical binding sites that attach the charged cell membrane, allowing for stronger attachment sites [35].

### 2.2. Natural Materials

It is known that native ECM components have nanoscale dimensions [36]; therefore, using nanostructured materials formed from collagen, gelatin, or fibrin hold promise for creating a successful scaffold. Incorporating nanofeatures is essential as they allow for a higher percentage of cellular attachment and availability of multiple focal adhesion points [37]. Because collagen is the most abundant extracardiac protein, developing scaffolds with this material should provide a physiologically relevant scenario when experimenting with CMs [38]. Furthermore, collagen types I and III make up the majority of scar tissue formed following an infarction [39]. Gelatin is a common natural material used in hydrogels for cardiac tissue engineering applications. It is derived from collagen, and can be chemically cross-linked with methacrylamide (GelMA), thereby reaching an adequate melting point to withstand body temperature [40]. Fibrin is a similar protein to collagen, but can degrade and be replaced by the ECM during wound healing [41]. In most cases, these materials are electrospun to form nanofibers which are then incorporated into the scaffold. However, careful consideration should be given to the drawbacks of these materials. They make for excellent in vitro scaffolds but can potentially contribute to fibrosis, degrade rapidly, and cause an immune response once used in vivo.

### 2.3. Nanotubes and Nanoparticles

Since the discovery of carbon nanotubes (CNTs), a vast amount of research has been conducted in exploring its potential use in the biomedical and biological fields. This material is known for its high aspect ratio, mechanical strength, light weight, superior electrical properties, and chemical and thermal stability [42]. The length of these tubes can range from hundreds of nanometers to micrometers while exhibiting a diameter of 0.4–2 nanometers [43]. For cardiac tissue engineering, both single walled carbon nanotubes (SWNTs) and multiwalled carbon nanotubes (MWNTs) are commonly used to create scaffolds with a prescribed elastic modulus and to promote CM electrical nature.

By using SWNTs and gelatin to create a composite scaffold as implants for Sprague–Dawley (SD) rats with large myocardial infarct, Zhou et al. [44] observed decreased left ventricular end-systole dimension, and restrained progress of left ventricle enlargement after four weeks of graft implantation. Their results suggest SWNTs increase the expression of intercellular adhesive junctions and electrochemical junctions, particularly N-cadherin and connexin-43 (Cx43); they attribute this result to the electrical properties of the SWNTs.

Interestingly enough, it has also been observed that MWNTs can selectively improve neonatal rat ventricular myocyte proliferation over that of fibroblasts in vitro [45]. Of those able to proliferate, healthy action potentials of single ventricular myocytes were recorded. Overall, the study suggests that CNTs prolong the proliferative state of undifferentiated cells and accelerate the maturation of differentiated CMs. These results elucidate the role of CNTs in creating the optimal environment for restoring heart tissue.

Shin et al. [46] also exploited the enhanced material properties CNTs have to offer but used GelMA for their scaffold. Their hybrid hydrogel had a compression modulus of 32 kPa (compared to 10 kPa in control) and decreased impedance. The results showed homogeneous and locally aligned CMs, and increased cell retention and viability. Developing a hydrogel with the correct stiffness is vital because, if the elastic modulus is too high, cardiac cells cannot properly mature or contract [47].

Gold is another material that has been found to promote electrical coupling between CMs. Literature has shown that by depositing gold nanoparticles onto coiled electrospun PCL fibers, superior electrical signal conductivity within the scaffold can be achieved [48]. Such results serve as evidence that the heart is naturally made up of fibers that stretch and recoil in the same fashion as CMs [49]. The results from using gold nanoparticles on the PCL fibers showed increased expression of *α*-sarcomeric actinin (a marker associated with cardiac muscle contraction), and reacted to significantly lower electrical fields compared to the control after seven days of culture. These observations show true promise since scaffolds must create the adequate contractile force to treat cardiac dysfunction [50].

### 2.4. Electrospun Scaffolds

Electrospinning is an inexpensive fabrication technique that has the capability of creating an interconnected pore structure and fiber diameters at the nanoscale [37]. Moreover, this method can accommodate a variety of synthetic and biological materials, making it an attractive technique for creating scaffolds. Kai et al. [51] electrospun PCL and gelatin to create a composite scaffold that mimics the aligned nanofibrous ECM of native heart muscle. They found higher cell attachment by CMs onto composite scaffolds as compared to pure PCL scaffolds. Furthermore, it was observed that CMs on aligned nanofibers were guided along the direction of fiber orientation, thus suggesting preference for anisotropic properties.

**Figure 3:**
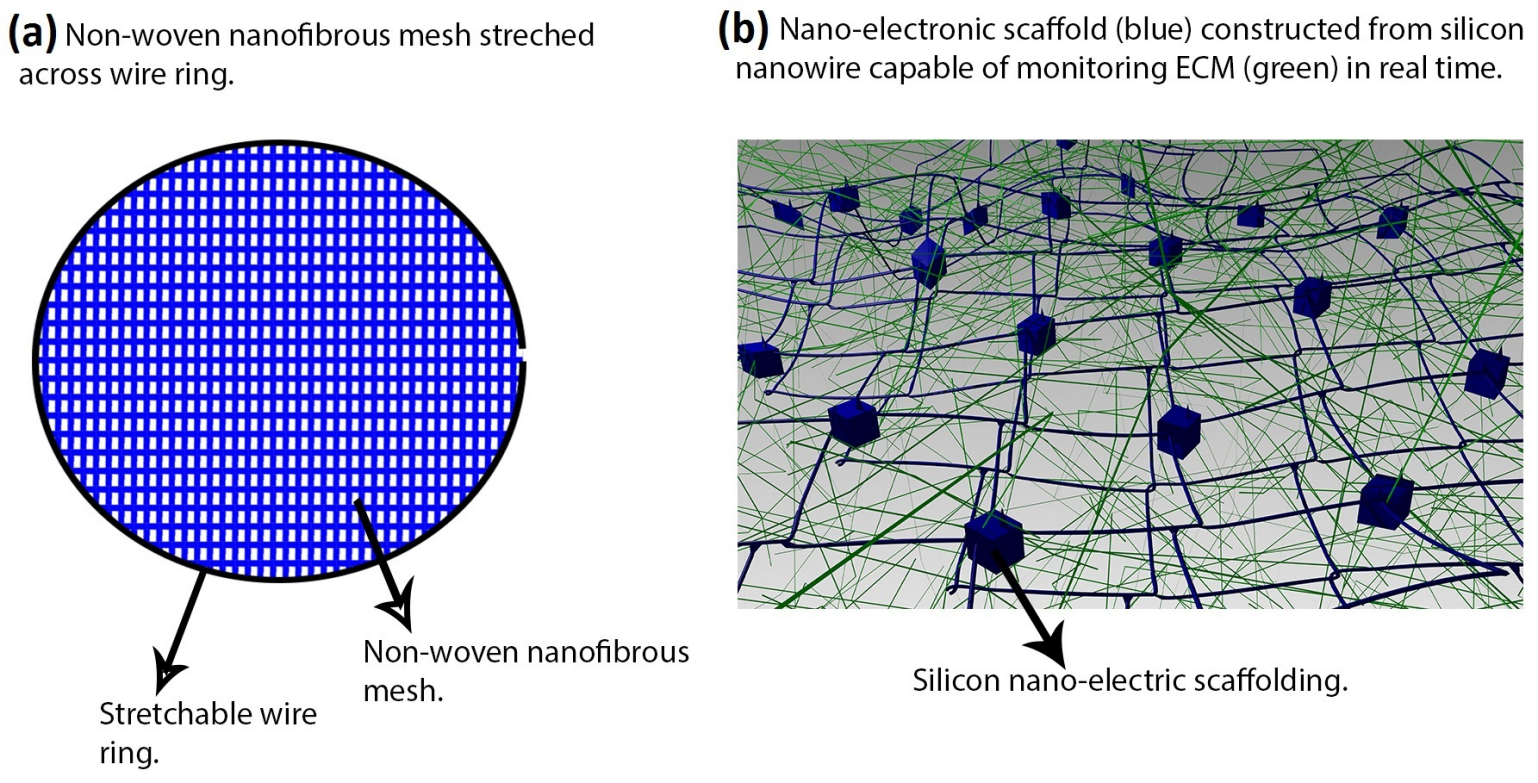
Two novel studies in cardiac tissue engineering: (**a**) electrospun polycaprolactone (PCL) nanofibrous mesh stretched across a wire ring used to create a passive load exerted by the wire allowing for cultured cardiomyocytes (CMs) to reach appropriate maturity prior to implantation [52]; (**b**) nanoelectronic scaffold (blue) constructed from silicon nanowire field effect transistors capable of real-time monitoring of local electrical activity within the extracellular matrix (ECM) (green) [55].

By using an electrospun PCL nanofibrous mesh stretched across a wire ring (Figure 3), Shin et al. created a passive load exerted by the wire that allows for cultured CMs to reach appropriate maturity prior to implantation [52]. This in vitro system attempts to mimic how native CMs are elongated and hypertrophied by mechanical loading; it resulted in contracting CMs after three days of culture and appropriate expression of actin, tropomyosin, and cardiac troponin-I.

Another study used electrospun poly(glycerol sebecate) (PGS) because of its elastic properties, large range of stiffness values, low cost, and hydrophilic fibers promoting cell adhesion and proliferation [53]. By injecting such material encapsulated with foreign CMs into the myocardial infarct region, they found favorable migration of native CMs onto the scaffold and increased expression of troponin-I and Cx43. An important observation by this study suggests the need to inject the scaffold onto both dead scar tissue and neighboring healthy tissue in order to promote the migration of primordial cells, thus easing regeneration [54].

### 2.5. Outlook

The vast majority of experiments testing scaffolds that incorporate nanomaterials have been executed in vitro, and have produced substantial evidence of favorable results, as noted by previously discussed studies. Doing so allows for controlled experiments, but proceeding to animal models would allow for testing in truly dynamic systems. It would be a significant advance in cardiac tissue engineering if there are consistent reports of in vivo nanomaterial enhanced scaffolds promoting adequate recovery in myocardial infarct animal models. However, laboratories often lack the resources to perform implantation of their scaffold. This calls for external support and collaboration between research groups to bring this field into fruition.

## 3. Patches

Another method used for regenerative cardiac tissue engineering is the implementation of a patch. Engineered cardiac patches for treating damaged heart tissues after a heart attack are normally produced by seeding heart cells within three-dimensional porous biomaterial scaffolds [56]. Cardiovascular patches can be divided into two groups: (i) conductive cardiovascular patches and (ii) non-conductive cardiovascular patches. The biomaterial used can be either biodegradable or non biodegradable [57].

These biomaterials are usually made of either biological polymers or synthetic polymers. These help cells to organize into functioning tissue; however, their poor conductivity limits the ability of the patch to contract as a single unit [56] so this calls for reinforcing the polymer patches with conductive nanomaterials. This is done using various methods; the most prominent are explained in the sections below.

### 3.1. Carbon Nanofiber Reinforced Patches

**Figure 4:**
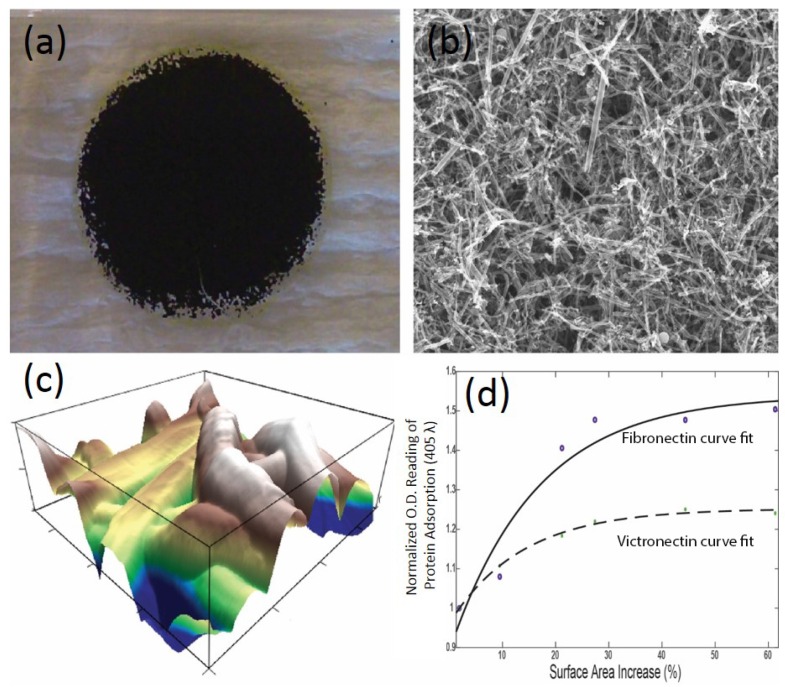
Cardiovascular patches embedded with nanomaterials play a key role in promoting stem cell growth around an infarct area of the myocardium: (**a**) the use of poly(lactic-co-glycolic) acid (PLGA) embedded with carbon nanofibers (CNFs) to generate a carbon nanofiber reinforced patch; (**b**) scanning electron micrograph of carbon nanofiber reinforced patch showing CNFs embedded in PLGA (at 10 K magnification); (**c**) atomic force micrograph of a carbon nanofiber reinforced patch depicting the topography of the patch; (**d**) using the data from atomic force micrographs to calculate the increase in surface area as one increases CNF concentration vs. protein adsorption, one can see that using CNFs can increase protein adsorption of a carbon nanofiber reinforced patch, thus increasing the possibility of cell recruitment and growth.

Because of their unique properties and exceptional physical and chemical characteristics, extensive research efforts are expended on utilizing carbon nanofibers (CNFs) in the field of tissue engineering (Figure 4). One study found that CMs grew more robustly by using carbon nanofibers embedded in PLGA than with conventional polymer substrates [57]. The same study tests various CNF to PLGA weight ratios and PLGA densities, but found that 50:50 PLGA to CNF at PLGA density of 0.025 g/mL best mimicked heart tissue tensile strength and conductivity. It also increased the absorption of proteins known to promote CM function.

Another study conducted by Kharaziha et al. [58] used carbon nanotubes embedded in GelMA. The conductive network formed by the CNTs in the porous gelatin media were the key characteristics of the CNT-GelMA, which lead to improved cell adhesion, organization and cell-cell coupling. There was also a high increase in the Young’s modulus after the addition of CNT, indicating a high increase in strength. About 3 mg/mL was shown to be the best composition of CNT, which showed a Young’s modulus of 30 kPa—more than double than that of plain gelatin [58].

### 3.2. Gold Nanofiber Reinforced Patches

Gold nanoparticles are in the limelight of cardiovascular tissue engineering as they are biocompatible, inert to cells and show localized surface plasmon resonance (LSPR) in the visible range. LSPR is an optical phenomenon arising from the interaction of the surface electrons with any electromagnetic radiation. This phenomenon causes both absorption and scattering components [59]. It plays a vital role in the conductivity of gold nanoparticles. Gold nanoparticles improve the electrical communication between adjacent cells. This enhances cell adhesion and proliferation [60]. Tal Dvir et al. developed a patch by dispersing three-dimensional nanowires in alginate that mimicked heart contractions, systolic, and diastolic pressures [56].

### 3.3. Outlook

From the studies shown above, it is evident that patches have great potential in becoming the primary treatment for end-stage heart failure. This novel approach to develop nanocomposites with spectacular material properties is an epitome of how engineering techniques can be used for biological applications to cure cardiovascular diseases.

## 4. Injectable Scaffolds

**Figure 5:**
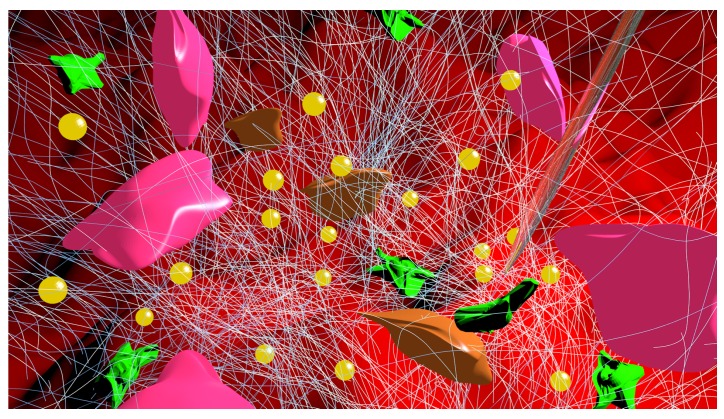
Following cardiac ischemic injury, lack of blood supply results in cardiomyocyte apoptosis (brown). After an inflammatory response, myofibroblasts and macrophages (green) migrate towards the injured area leading to fibrosis thus creating scar tissue (white). When scaffolds are applied to the injured area (in this case by injection via syringe (clear)), exploiting the superior material properties of nanomaterials such as gold nanoparticles (yellow) have demonstrated reduced scarring and often slowed left ventricular remodeling. The (pink) cardiomyocytes in this figure are shown to regenerate along the site of scaffold injection.

The main appeal behind injectable scaffolds is their simplification or even replacement of invasive surgery because they can be inserted via syringe or catheter. The benefits of this procedure can readily be seen through anesthetics, vaccinations, and anti-inflammatory injections; therefore, if an injectable scaffold that can regenerate the infarct zone following a heart attack can be developed, this technology has the potential of revolutionizing cardiac tissue engineering (Figure 5). However, drawbacks to consider inherently include less control of degradation rates, scaffold shapes, and pore distribution as compared to conventional scaffolds due to the flow of the material into its destination [61]. Nonetheless, progress has been made in developing injectable scaffolds for cardiac tissue engineering.

One approach to creating an injectable scaffold is through self-assembling peptides. These peptide sequences can assemble into a nanofibrous hydrogel once injected into the infarct region and have been shown, in a porcine model, to increase interventricular septum thickness and prevent ventricular remodeling [62]. The same study also reports that use of this nanofibrous hydrogel increased retention of transplanted autologous bone marrow mononuclear cells and their differentiation into endothelial cells and smooth muscle cells, suggesting the favorable biomimetic properties of the scaffold [62].

Another common method to create an injectable gel is from decellularized ECM. For example, porcine myocardial tissue was extracted and decellularized so that it could form a gel in vivo after being passed through a catheter tube [63]. The gel maintained its biochemical composition, re-assembled to form a nanofibrous scaffold, and promoted vascular cell infiltration during in vivo experiments. The nanofibrous characteristic allows for high surface area to volume resulting in better cell adhesion, but some of the gel’s 3D structure is lost when conforming the gel to an injectable.

Reverse thermal gels have also been used to create injectable scaffolds; they have the capability of undergoing phase transition triggered by a temperature change. Other hydrogels normally require cross-linkers or UV radiation to prompt gelation, thus reverse thermal gels offer a simple alternative [64]. Wang et al. demonstrated their potential by using oligo[poly(ethylene glycol) fumarate] (OPF) as an injectable hydrogel seeded with mouse embryonic stem cells (mESCs) to treat myocardial infarction in a rat model [65]. By using the model’s own body temperature, the gel can form in situ within a given time, thereby simplifying the process of incorporating cells and growth factors into the gel prior to injection. Their study reports a significant amount of the OPF hydrogel 24 h after injection and a 10% improvement of mESC presence in the infarcted area using OPF as compared to the control (injection of only mESCs).

Instead of using only a scaffold to regenerate tissue, Paul et al. [66] used graphene oxide nanosheets complexed with pro-angiogenic human vascular endothelial growth factor-165 plasmid DNA (DNA_VEGF_) to create a GelMA hydrogel capable of being injected into cardiac tissue and retain the necessary mechanical strength to withstand the stresses generated in vivo. Slow release of the DNA_VEGF_ served as therapeutic effect to the infarct region, and over the course of three days showed no signs of toxicity or inflammation. Their procedure resulted in myocardial vasculogenesis ultimately leading to reduced scar tissue and improved heart function after seven days of injection within their rat model [66].

Injectable materials have the potential of becoming the paradigm technique for cardiac tissue engineering due to its facile application, but there are still considerable roadblocks to overcome such as proper guidance to the infarct zone and control of final scaffold geometry upon arrival. Although the studies reviewed here report positive results, there is innate hesitation to proceed with human testing because of the inability to control the injectable material through the blood stream in real time. Considerable advance in understanding the fluid and soft material mechanics under physiologically relevant conditions of this procedure is the subsequent step for this technique.

## 5. Miscellaneous Approaches

Apart from constructing scaffolds that guide tissue regeneration, it is also important to monitor the state of cells in real time. Tian et al. [55] proposed a nanowire nanoelectronic scaffold with the capability of monitoring single CM contraction at corresponding silicon nanowire field effect transistors (Figure 3). This technology can potentially be implemented in scaffolds of varying materials to find the optimal material for CM scaffolding by comparing CM behavior.

Injectable scaffolds seek to create a 3D environment for tissue regeneration; however, another common approach is injection of purely nanomaterials to serve as delivery vehicles to provide therapeutic effects to the infarct region. Nanoparticles can be administered through intravenous injection and are then guided to the infarct zone by the enhanced permeability and retention (EPR) effect—a condition in which the vasculature is disrupted [67]. Using this principle and their ability of flowing through obstructions due to their smaller size, dodecaluoropentane (a type of perfluorocarbon with higher electrophilic fluorinated bonds) nanoparticles were used as an oxygen carrier [68]. The tudy showed a significant reduction in infarct size compared to their control using mice models.

Another study also took advantage of the EPR effect, but looked at using nano-sized PEGylated liposomes to carry therapeutics and selectively bind to the overexpressed angiotensin II type 1 receptor in the infarcted heart [69]. The nanoparticles were injected through the right jugular vein and were shown to successfully remain at the infarcted region. These reports serve to show the potential of nanoparticles as drug delivery systems to be used in combination with 3D scaffolds.

## 6. Toxicity

When investigating nanomaterials, toxicity must be addressed. Toxicity has widely been defined as the amount of substance that can kill an organism, and may also refer to the effect on the organism as a whole or its infrastructure [70]. The toxicity of the material at the cellular level is called cytotoxicity—apoptosis and necrosis are two particular sublethal markers of cytotoxicity [71,72].

Nanotoxicity is a field that studies the effect of novel nanomaterials on living species. This field has recently emerged due to the extreme difference in behavior of nanomaterials as compared to their bulk counterparts [73]. The factors that affect the interaction of nanomaterials with biological systems are size, shape, surface area, chemical composition, lattice structure, surface chemistry, surface charge, and aggregation state [72].

Overall, carbon-based nanomaterials (CBNs) are some of the most attractive nanomaterials because of their spectacular properties at the nanoscale; however, studies have shown that their interaction with organisms can be highly toxic. CBN’s have been shown to aggregate in the combustion streams of fuel gas and air, which is then inhaled by humans on a daily basis, often times causing lung related problems [70,74,75]. A recent study conducted by Arnaud et al. [75] showed that CBNs lead to proliferation inhibition and cell death. It also concluded that CNTs are less toxic than CNFs, and that CBNs are more toxic when surface chemistry changes, such as adding carbonyl compounds to the surface, are made. A study conducted by Savi et al. showed that titanium dioxide nanoparticles enhanced the susceptibility of adult rat cardiomyocytes to cardiac arrhythmia via shortening of re-polarization time [76]. Nanomaterials have greatly contributed to society in terms of technological advances, but more research must be conducted on the adverse effects of their toxicity and prevention of cytotoxicity [73].

Toxicity studies seem to show paradoxical results; however, the dosage of CBNs must be taken into consideration. Furthermore, most experiments were conducted in two-dimensional systems, but the human body is a three-dimensional dynamic system. The amount of CBNs in terms of weight and dosage used to develop patches, scaffolds and injectables are far less compared to the size of the human body. In addition, the toxicity of the CBNs can be tackled by functionalizing them. Therefore, toxicity may not be as concerning as it seems. Hence, nanomaterials still have great potential in revolutionizing treatment techniques for CVD.

## 7. Conclusions

Heart transplantation is a successful way to treat end-stage heart failure, but alternative treatments should be developed to reach a larger number of patients. Additionally, to address the problem of post heart attack left ventricular dysfunction, scaffolds, patches, and injectables exploiting the superior characteristics of nanomaterials are a potential therapy for tissue myocyte engineering (Table 1). Employing synthetic materials in scaffolds usually allows for easier characterization of material properties, but does not reproduce the native ECM. Creating a nanorough surface with synthetic materials has shown to increase cell viability and proliferation. Moreover, creating a composite material from a synthetic and CNTs or gold nanoparticles has become a popular approach. CNTs and gold nanoparticles are typically used to enhance the mechanical and electrical properties of the scaffold, thus replicating the native myocardium.

In the meantime, there are still some lingering questions to be addressed and potential future research avenues. Nanomaterials enhance CM expression of important markers, but the fundamental mechanisms underlying this phenomenon are unknown. Nanomaterials also promote cell adhesion, but perhaps deeper understanding of the substrate-cytoskeletal dynamics could provide ideas for improved material manipulation. Much of the recent experiments have been done in vitro, therefore validating a scaffold’s non-immunogenic property is crucial. In all, cardiac tissue engineering has progressed dramatically in the past two decades, but much work lies ahead before CVD can efficiently be addressed, and before these techniques can be successfully applied to improve ventricular function and prevent heart failure in myocardial infarction patients.

## Figures and Tables

**Table 1:** To address the problem of post heart attack left ventricular dysfunction, scaffolds, patches, and injectables exploiting the superior characteristics of nanomaterials are a potential therapy for tissue myocyte engineering.

Nanomaterials for CVD						
Material	Type	Category	Model	Nanofeature	Results	Reference
PEG	Synthetic	Scaffold	in vitro	Nanopillars	Enhanced cell binding cites increasing CM adhesion	[22]
PGA	Synthetic	Scaffold	in vitro	Nanofibrous	Higher cell viability due to increased hydrophilic properties	[27]
PPy/PCL/Gelatin	Synthetic/Natural	Scaffold	in vitro	Nanofibrous	Stronger attachment sites due to conductive PPy	[35]
SWNT/Gelatin	Carbon/Natural	Scaffold	SD Rats	Nanotubes	Decreased left ventricular end systole dimension	[44]
CNT/GelMA	Carbon/Natural	Scaffold	in vitro	Nanotubes	Biomimetic elastic modulus and decreased impendance increased cell retention	[46]
AU/PCL/Gelatin	AU/Synthetic/Natural	Scaffold	in vitro	Nanoparticles	Enhanced functional assembly and increased Cx43 expression	[60]
PCL/Gelatin	Synthetic/Natural	Electrospun	in vitro	Nanofibrous	Aligned nanofibers guided cell orientation suggesting CM preference for anisotropic properties	[51]
PCL	Synthetic	Electrospun	in vitro	Nanofibrous	Stretched scaffold using a wire ring to promote CM maturity to increased troponin-I expression	[52]
Peptides	Natural	Injectable	Porcine	Nanofibrous	Increased interventricular septum thickness and slowed ventricular remodling	[62]
Decellularized ECM	Natural	Injectable	Porcine	Nanofibrous	Promoted vascular cell infiltration at the infarct zone	[63]
PLGA-CNF	Carbon/Natural	Patch	in vitro	Nanofibrous	Increased mechanical strength, conductivity and enhanced cardiomyocyte growth	[57]
CNT/GelMA	Carbon/Natural	Patch	in vitro	Nanofibrous	Improved cell adhesion, organization and cell-cell coupling	[58]
AU/Alginate	Gold/Natural	Patch	in vitro	Nanofibrous	Improved electrical communication between cells resulting in enhanced cell adhesion and proliferation	[56]

## Abbreviations

The following abbreviations are used in this manuscript:

AU	Gold
CBN	Carbon Based Nanomaterials
CM	Cardiomyocyte
CNF	Carbon Nanofibers
CNT	Carbon Nanotube
CVD	Cardiovasular Disease
Cx43	Connexin 43
EPR	Enhanced Permeability and Retention
FDA	Food and Drug Administration
GelMA	Gelatin Methacrylate
LSPR	Localized Surface Plasmon Resonance
LVAD	Left Ventricular Assist Device
mESC	mouse Embryonic Stem Cells
MWNT	Multi-walled Carbon Nanotube
OPF	Oligo[poly(ethylene glycol) fumarate]
PEG	Poly(ethylene glycol)
PGA	Poly(glycolic) acid
PCL	Poly(caprolactone)
PLGA	Poly(lactic-co-glycolic) acid
PLA	Polylactic acid
PPy	Polypyrrole
PG	Poly(glycerol sebecate) and gelatin
PGS	Poly(glycerol sebecate)
RNT	Rosette Nanotubes
SD	Sprague–Dawley
SWNT	Single-walled Carbon Nanotube
